# The impact of a supplementary medication review and counselling service within the oncology outpatient setting

**DOI:** 10.1038/sj.bjc.6603634

**Published:** 2007-02-20

**Authors:** H Read, S Ladds, B Rhodes, D Brown, J Portlock

**Affiliations:** 1 Department of Pharmacy, Worthing and Southlands Hospitals NHS Trust, Lyndhurst Road, Worthing, West Sussex BN11 2DH, UK; 2 School of Pharmacy and Biomedical Sciences, St Michael's Building, White Swan Rd, Portsmouth, Hampshire PO1 2DT, UK

**Keywords:** pharmacy, technician, medication review, breast cancer, controlled trial

## Abstract

The impact on the care of breast cancer patients, of a pharmacy technician-led medication review and counselling clinic, provided in an outpatient setting, was investigated using a controlled randomised study. Compared to the controls, clinic patients showed a significantly improved level of understanding of their chemotherapy support medication (95% CI for difference in mean knowledge rating scores=2.165–2.826, *P*<0.001) and a significant reduction in the median number of support items required (two compared to five in the control, *P*<0.001). This resulted in a significant reduction in mean medication expenditure per patient (£26.70 *vs* £10.20, 95% CI for the mean difference in cost £6.72–£26.26, *P*<0.001). The clinic was also associated with significant reductions in chemotherapy delays (*P*<0.001) and dose reductions due to side effects (*P*=0.003). Other benefits from the clinic were a reduction in pharmacy dispensing time and a highly significant reduction in pharmacy time spent resolving post-clinic prescription queries (*P*<0.001). Taking into account the initial technician training cost, the scheme represented an annual saving to the Trust of over £15 000. The clinic serves as a model for those wishing to improve outpatient services to breast cancer patients.

It is widely accepted that a cornerstone of effective breast cancer chemotherapy is to maximise the appropriate dose while minimising the often unpleasant and debilitating side effects. A recent study in women receiving chemotherapy in the first 12 months after diagnosis for breast cancer showed that the incidence of serious effects requiring emergency intervention in hospital (61%) is likely to be higher than that predicted from clinical trial data (Hassett *et al*, 2006). While support medication strategies have improved over the years, it is not uncommon for patients to become reluctant to continue with further cycles of treatment if they have already experienced side effects from initial cycles of chemotherapy (Craig and Powell, 1987; Atkins and Fallowfield, 2006). Chemotherapy support medication is co-prescribed with the intention of minimising side effects with the hope that this will improve chemotherapy concordance, which in turn will assist the success of chemotherapy.

Research has indicated that cancer patients who concord with oral chemotherapy regimens have a higher survival rate than those who do not (Porzsolt *et al*, 1989; Spiegel, 1997).

Concordance can be described as a process of prescribing and medicine taking in a prescriber – patient partnership (Stevenson, 2001). This is particularly important in situations like chemotherapy support medication, where the patient must first recognise the side effect of the chemotherapy, then select the correct drug and self-administer the right dose for the correct period, remote from her immediate healthcare team. All members in the healthcare team have a responsibility to foster concordant relationships with patients. Prescribing physicians may not be able to do this for a variety of reasons (Stevenson *et al*, 2000). These include lack of time to discuss individual medication issues such as potential side effects (what to look out for and what to do about them) and because the prescribing setting may not lend itself to effective counselling.

Cancer patients face the challenges of understanding often complex and protracted chemotherapy regimens, their side effects and management, while at the same time coming to terms with a potentially life-threatening diagnosis. Acceptance of treatment is largely based on the reality of a terminal condition without it and patient understanding of how chemotherapy can be made less debilitating with support medication is fundamental to building a concordant relationship.

Pharmacists and more recently, pharmacy technicians counsel patients on their medications in a variety of settings as part of routine clinical pharmacy practice. Patients may have good access to staff on the ward where they receive their chemotherapy, but a busy ward frequently is not conducive to effective information transfer. Studies conducted by pharmacists in other disciplines, such as anticoagulation (Radley and Hall, 1994; Boddy, 2001), dermatology (Tucker, 2004) and palliative care (Austwick *et al*, 2002) which showed improved quality of care, patient understanding and use of resources, suggest that the outpatient clinic may be a suitable alternative setting.

Like many breast cancer clinics, patients are seen at Worthing Hospital under a shared care agreement with another local NHS Trust. A co-ordinated, multi-disciplinary team approach relies for its success on good communication between clinicians, chemotherapy nurses on the Medical Day Case Unit (MDCU) and pharmacists and pharmacy technicians working in aseptic services. Prior to this study, any pharmaceutical issues surrounding their support medication could only be resolved post-clinic, when the physician had departed. It was suggested that the service, which was under increasing capacity pressures, could be improved by direct pharmacy involvement at clinic level, when the patient and all healthcare team members were present.

A pharmacy technician who has successfully completed an accredited medicines management course is deemed to be competent in routine medication review and drug history taking. These are useful attributes when educating patients on their chemotherapy support medication and assessing their ability to cope with this. This then frees the pharmacist to deal with more complex pharmaceutical care issues raised by the latter, such as drug interactions and side effects, thus making best use of pharmacy resources.

As a result, a pharmacy technician-led outpatient clinic for breast cancer patients being treated on MDCU at Worthing Hospital was proposed. This was considered a potentially useful setting in which to perform a complete medication review and research its effect, through a controlled study on a range of features of patient care.

## METHODS

Approval for the study including all data collection forms and methods, was obtained from the West Sussex Local Research Ethics Committee (04/Q1911/45) and the Sussex NHS Research Consortium (0457/WASH/2004) on 23/11/04 and 6/1/05 respectively, prior to commencement of the study.

### Technician training and accreditation

The research technician (BR) underwent the South East Medicines Management Education and Development Team's accredited medicines management course. The course consists of private study, supplemented with study days and is assessed by demonstration of competence in work-based activities in a written portfolio and observed structured clinical examination. This provided a knowledge and skills framework that was supplemented by additional directed study on patient counselling, chemotherapy regimens for breast cancer and relevant support medication, identification of side effects and drug interactions. Assessment of the pharmacy technician's knowledge of chemotherapy support medication was conducted by formal written examination. Assessment of the technician's counselling skills was undertaken by in-practice observation following completion of a Trust in-house counselling course.

All assessment and training was undertaken during one year (February 2004 to January 2005). Following assessment, the technician was established within the outpatient clinic in a dedicated consulting office. Approval for this was obtained from the consultant oncologist treating breast cancer patents at Worthing Hospital and the senior nursing staff on MDCU and in the Outpatient Department.

### Study design

**Figure 1:**
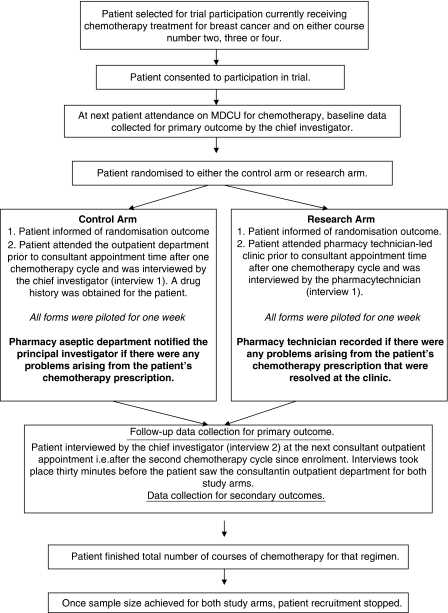
Overview of the research design.

The research took the form of a randomised, controlled study, outlined in Figure 1. The project lasted 12 months including a 1-week pilot study consisting of ten patients to assess applicability of procedures and data collection forms. The pilot indicated that no appreciable changes to protocol or data collection procedures were required and the ten pilot patients were included in the overall analysis.

### Patient recruitment

Patients with a diagnosis of breast cancer and on course 2, 3 or 4 of their chemotherapy, as identified from their records, were approached by the chief investigator (HR) on attending MDCU. Each patient was provided with verbal and written information about the research and asked to provide written consent. Patients receiving treatment for either adjuvant or metastatic disease receiving a variety of regimens, were included. Patients under 18 years old were excluded.

Consenting participants were informed that on their next MDCU visit, they would be asked a series of questions testing their understanding of their support medication.

### Patient randomisation

Patients were randomised to either the control arm, where they received conventional care, including routine ward counselling from a nurse on the use of their support medication prior to discharge, or to the experimental arm where they attended the pharmacy technician-led outpatient clinic. Randomisation was by a computer-generated random number table produced in Microsoft Excel. Each patient received an information sheet explaining which study arm they had been allocated to and what this involved.

### Patient assessment at follow-up

Patients were interviewed by the chief investigator at their next outpatient appointment after a further chemotherapy cycle (usually of 3 weeks’ duration) had been completed.

### Control arm

Prior to their next visit, subjects were sent a letter inviting them to arrive early at the clinic and to bring a copy of their GP repeat medication list with them. At the clinic and prior to their scheduled consultant appointment, they were also interviewed about this list and any other medications they were taking in addition to their chemotherapy. The information was used to compile an accurate drug history and to highlight any potential drug interactions with the patient's chemotherapy or chemotherapy support medication. Any problems so identified were forwarded to the consultant to discuss with the patient when they saw them at the allocated time.

The prescription for the patient's next cycle of chemotherapy resulting from the consultation was tracked via the pharmacy aseptic department. Any issues that arose from this prescription were resolved by that department's personnel and recorded as part of the study. This was primarily to document the time taken to resolve prescription problems by contacting the consultant after the clinic.

At their subsequent clinic appointment, patient understanding of their support medication was re-assessed as at baseline.

### Research arm

Patients were asked to attend the pharmacy technician-led outpatient clinic prior to seeing the consultant oncologist at their next outpatient appointment clinic. At the pharmacy technician-led clinic, the following issues were recorded:

1. A full drug history
2. Potential drug interactions
3. Whether supplies of support medications were required
4. The patient's perception of the clinic and whether they found it beneficial or not.

Information from items 2 and 3 was noted and attached to the patient's notes so that the consultant could make use of it when consulting with the patient.

After the consultation, the resulting prescription was tracked as in the control arm.

Patients were interviewed again by the chief investigator prior to their next consultant appointment, to assess their knowledge of support medication. As with the control arm, this took place after two chemotherapy cycles had elapsed since enrolment. The chief investigator remained unaware of which arm of the trial each patient was in.

### Primary outcome measure – baseline assessment of patient understanding of support medication

The chief investigator (HR) interviewed each consented patient using a standard question set, asking the patient to explain what each of their support medications was for and how they used it. Assessments were recorded as an overall rating of the subject's understanding on a Likert scale from 1=very poor to 5=very good, plus a drug-specific score derived from marks awarded for various pieces of information: drug name (+2); indication (+2); dose frequency (+2); dose duration (+1); dose (+1). Thus the maximum score per drug was 8 and the minimum 0. The average score per drug was also calculated. Assessments were conducted prior to randomisation; the chief investigator was blind during subsequent, post-randomisation assessments.

### Secondary outcome measures

There were a number of secondary outcomes of interest. All secondary outcome measures were compared between control and clinic arms directly.

1. The numbers of patients experiencing delays in receiving their chemotherapy.
2. The numbers of patients requiring chemotherapy dose reductions.
3. Numbers of patients having potential drug interactions between GP prescribed medication and chemotherapy or chemotherapy support medication.
4. The number of chemotherapy support medication items supplied per patient.
5. The average cost of chemotherapy support medication supplied per patient.
6. The numbers and costs of chemotherapy support medication not supplied.
7. The average amount of pharmacy time per patient spent resolving medication issues.
8. The numbers of patients requiring a prescription intervention at the point of dispensing.

All medication costs were calculated using hospital prices prevailing at the time of the study.

### Data analysis

All statistical analyses were conducted using Minitab version 13 (Minitab Inc., State College, PA, USA), taking *P*<0.05 as an acceptable level of probability indicating statistical significance.

For the primary outcome measure (patient knowledge score) mean scores were compared before and after clinic attendance in both the control and clinic arms using Student's *t*-test.

For the secondary outcome measures, the proportions of patients experiencing chemotherapy delays and reductions were compared using the *χ*^2^ test.

The numbers of chemotherapy support medications supplied for each patient were compared using the Mann–Whitney *U*-test and the mean costs of items supplied were compared using a two-sample *t*-test. The mean times spent resolving prescription issues in the two study arms were compared using a two-sample *t*-test.

## RESULTS

All the chemotherapy regimens encountered in this study were prescribed against standard proformas, used on a day to day basis across the Sussex Cancer Network; these had been checked and verified across the whole cancer network, and were last scrutinised as part of the National Cancer Peer Review Programme in 2005/6. Antiemetics were also prescribed according to the cancer network guidelines on the prevention and treatment of nausea and vomiting in adults receiving chemotherapy. The prescribing of support medication was absolutely consistent across the two arms of the trial as the same standard network chemotherapy proformas were used.

Chemotherapy regimens varied between patients according to disease stage and comorbidity. Patients were not stratified during randomisation for the nature of the chemotherapy regimen used or cycle of therapy. Actual regimens and cycle numbers for control and clinic arms are shown in Table 1; there was a significantly higher proportion of patients receiving adjuvant therapy in the clinical arm compared to the control (*P*=0.003, *χ*^2^=9.074). There were no differences in cycle numbers. The imbalance between the two arms of the trial, in the numbers of patients receiving trastuzumab monotherapy, is discussed below.

One patient withdrew from each arm of the study, so the base in each was reduced to 69. Key results are summarised in Table 2.

### Primary outcome: level of patient understanding about support medication

There were no statistically significant differences between the mean baseline scores in the control and clinic arms (*P*=0.942) or between second measurement and baseline in the control arm (*P*=0.822). In contrast, the difference from baseline in the clinic arm was very highly significant (*P*<0.001).

### Secondary outcome measures

Significantly fewer patients experienced chemotherapy delays after attending the pharmacy technician-led clinic (*P*=0.001) and significantly fewer patients also experienced chemotherapy dose reductions (*P*=0.003).

Forty-seven (33.8%) of the 139 patients eligible for baseline evaluation had potential drug interactions highlighted. The difference between the control and clinic arms was not statistically significant (*P*=0.188); these data suggest that the pharmacy technician-led clinic detected more potential drug interactions but this hypothesis is unproven. Most drug interactions were between GP prescribed medication and chemotherapy support medication rather than the chemotherapy itself. The most common interactions were between corticosteroids and non-steroidal anti-inflammatory drugs, antidiabetic agents, antiepileptic agents and antihypertensive medication. In the majority of cases it was accepted that a short course of corticosteroids would not lead to a clinically significant interaction with these other agents; however, it would be unwise to assume that this would be the case in all patients where polypharmacy and comorbidities may mitigate.

There were significantly fewer chemotherapy support items supplied per patient in the clinic arm compared to the control (*P*<0.001); this cross-correlated with additional data from the pharmacy technician-led clinic where the median number of items not supplied was also three. The bulk of unnecessary prescribing consisted of antiemetics and mouth care products; G-CSF was also prescribed unnecessarily in a small number of patients. A pilot study had shown that it took an average of 3.6 min for a pharmacy technician to dispense and a pharmacist to check an item of chemotherapy support medication; this represented a time saving of approximately 10.8 min per breast cancer patient in this study. If the over-prescription at the first cycle was perpetuated over the full five courses of therapy, then a total of 54 min of pharmacy time would be wasted. In terms of dispensary time saved, this would amount to approximately 146 h per annum.

There was a highly significant reduction in mean cost of items supplied to control patients compared to clinic patients (*t*=3.34, point estimate for difference=16.49, CI=6.72–26.26, *P*<0.001). Information collected in the research arm allowed calculation of a mean cost of medication not supplied of £17.54 per patient. Again, if over-prescription was perpetuated over five courses, this would be a waste of £87.70 and projecting over a typical year involving 162 patients, a saving of £14207.40 could be made.

Technician time invested overall in the clinic amounted to 24.7 h per annum, costing £305.29 (based on current salary scales). This input was calculated by subtracting the time saved not having to resolve prescription-related problems post-clinic from the actual time expended in the clinic. In addition, the technician time saved by not dispensing unnecessary items was 146 h per annum, costing £1804.56. Therefore, the total time saved was 146–24.7=121.3 h per annum, saving £1499.27.

The above savings should be set against the cost of the initial technician training in medicines management (approximately £1800); but this is a one-off investment paid for by the Strategic Health Authority and a mandatory requirement of standard post-qualification training for all pharmacy technicians in the Trust. The additional costs incurred in preparing the technician for the cancer clinic role were £155 – again a one-off investment.

The mean cost of items wasted from previous supplies returned to the pharmacy by patients in the clinic arm of the study and subsequently destroyed was £26.70. As an indication of unnecessary wastage this would amount to an annual saving of £4325.40, based on the throughput of breast cancer patients at Worthing Hospital. This is probably a minimum, as patients do not always return unwanted or unused medicines to the pharmacy.

There was a highly significant reduction in the amount of pharmacy time per patient spent resolving prescription issues in the clinic arm compared to the control arm of this study (*t*=5.31, point estimate for the difference=8.0, 95%CI=5.05–11.04, *P*<0.001); fewer patients required a prescription intervention at the point of dispensing in the clinic arm compared to the control arm (30 *vs* 45.7%) but the difference did not reach statistical significance (df=1, *χ*^2^=3.439, *P*=0.064); but together, these results suggest that those interventions required in the clinic patients were of a more complex nature.

## DISCUSSION

The pharmacy technician-led clinic for breast cancer patients could easily be developed for other cancer specialities where routine chemotherapy support medications are used. The hospital clinic is a suitable environment for such exercises. While no other similar published reports could be found, the benefits of medication review clinics in other specialities have been noted. Morlidge (2001) reported upon the introduction of a renal medication review clinic that showed similar benefits, such as identification and withdrawal of unnecessary drugs, avoidance of unnecessary repeat medication, improved concordance with therapy, better drug history taking and reductions in wastage of drugs and pharmacy time. Running the clinic alongside the consultant clinic facilitated prompt liaison and ensured medication problems were promptly resolved. Austwick *et al* (2002) reported the results of a study in palliative care, where drug regimens can be as complex as those in cancer chemotherapy; it was shown that the pharmacist's input into the outpatient clinic proved invaluable. In general, patients left the pharmacy clinic with a better understanding of their medication as well as knowing how to combat unpleasant side effects. Supply of medication was more efficient, drug histories were kept up to date and medication problems were better addressed.

### Patient knowledge

After their initial diagnosis, breast cancer patients at Worthing Hospital receive an explanation of their chemotherapy treatment and its potential side effects from specialist nursing staff on MDCU. Patients then receive their first cycle of chemotherapy after which they are given their chemotherapy support medication with a one-off explanation of how, when and why each item should be taken. This study has shown that patients who attend the pharmacy technician-led clinic had a significantly better level of understanding of their chemotherapy support medication compared to control patients receiving best standard care.

Pharmacy and nursing staff play key roles in educating patients about their medicines; both are equipped with specialist knowledge needed to relay important treatment information to patients. Exploration of the factors that may have influenced the success of the pharmacy technician-led clinic is worthwhile.

Chemotherapy is complex. The stigma surrounding cancer and its treatment can understandably be very disheartening for patients who after diagnosis, receive a plethora of important information about their new (to them) condition. Some of this is re-emphasised, often at the patient's request, during the first chemotherapy session on the ward. Apprehension and the chemotherapy itself can often leave the patient feeling very tired and confused. Anecdotally, patients at the pharmacy technician-led clinic voiced the opinion that an environment away from the ward was more relaxing and that in the ward environment, their concentration had declined significantly before they received information on their chemotherapy support medication. In contrast, the pharmacy technician-led clinic provided an atmosphere where they felt able to ask questions about their support medication and absorb the information. The co-ordination of pharmacy and nursing input may therefore be a crucial step in improving this aspect of care.

Many different aides memoir or treatment prompts were observed to be used by patients in this study. These ranged from handwritten notes on the outer cartons to complete computer spreadsheets allowing dose administration records to be kept. Patients with more support medications relied heavily on these treatment prompts and it was observed that in general, those without prompts, particularly those taking larger numbers of support medications, knew the least about their medications. These patients scored poorly on the knowledge rating scale; this was undoubtedly exacerbated by the fact that their mean score was computed from a high number of treatments.

The scoring system used in this study was subjective but applied consistently by one person (the chief investigator). Its application in practice allowed simple rules to be used to assist consistency. The scale was designed to allow weighting of parameters depending on their importance to the patient's understanding of their treatment with more emphasis on drug name, indication and frequency than strength and duration. Interestingly, the most common information omitted by patients during interview was indication and strength. These two pieces of information are very useful, but for different reasons. While the former is obviously important when the patient has to select the appropriate support medication for symptom relief from a range of medicines, the strength is of importance when supplying the information to a healthcare professional so that a further supply or appropriate substitute can be organised. The scale also demonstrated consistency over time. In the control arm, there was little variation in baseline and follow-up scores (see Table 2).

In general, patients tended to rate their level of understanding of support medication higher than the investigator at both stages of the study. This was the first question they were asked during the interview. Following the assessment questions, many were surprised at how little they knew. Self rating may have been lower if the initial question had been asked at the end.

Whatever the patient's initial level of understanding about their support medication the study has demonstrated that the pharmacy technician-led clinic produced significant improvements. Work from a wide range of disciplines and clinical settings, indicates that improved knowledge about their medications is linked to improved patient concordance with therapy; although it seems difficult to demonstrate beyond doubt. Horne (1998a) cites evidence for a direct link between adherence to medication and perceived symptomatic benefit; in our case, improved control of the side effects of cancer chemotherapy. Clearly the pharmacy technician-led clinic is an excellent opportunity to impart benefit information.

Horne (1998b) undertook an extensive review of the literature surrounding the factors affecting adherence. He found that although a basic awareness of how and when to take medicines was one pre-requisite for adherence it was insufficient on its own; other factors such as the patient's general health beliefs and specific beliefs about medicines also played an important part; furthermore, patients who were more satisfied with their care were more likely to adhere to their treatment regimen. More specifically, Craig and Powell (1987) observed that effective management of chemotherapy-induced emesis improved overall patient adherence and enhanced the effectiveness of patients’ chemotherapy. The pharmacy technician-led clinic in our study provided the opportunity for a full discussion on the support medication in the context of the patient's primary cancer chemotherapy, both in terms of symptom recognition, choice of support medication and clarification of dosage instructions. Patients were also given the opportunity to ask questions about their management.

Breast cancer and its treatments involve many different facets for the patient to get to grips with. Horne (1998b) has suggested that in complex diseases, a patient who receives the information over time in a variety of appropriate settings is likely to absorb more and be a more concordant, happier and hopefully healthier patient. A one-off session at the start of treatment is unlikely to be as effective as successive encounters, where the patient can discuss progress and have new enquiries dealt with (Horne, 2000). This supports the repeat use of the pharmacy technician-led clinic on each visit to the breast cancer clinic.

For most cancer patients, acceptance of chemotherapy is based on the reality of a potentially terminal condition without it. Once this initial step has been taken, information on how to modify the debilitating effects of chemotherapy is fundamental to building a successful concordant relationship based around the patient's needs. The healthcare team need to work on this concordant relationship with the patient by providing excellent support that engages in activities that maximise benefit from the patient's perspective.

It has been demonstrated that more active listening by healthcare professionals is needed to avoid missing important verbal and non-verbal signals that need to be followed up with the patient (Blenkinsopp, 2001). The successful delivery of information must include an interactive discussion with the patient. It has been suggested that non-concordance may stem not from lack of understanding but rather a conscious decision by a patient not to take his/her medication as directed. The reasons for this are undoubtedly multifactorial but must surely include the fear of medication side effects. Once they have accepted their diagnosis and the need for chemotherapy and have recognised the side effects of the latter, cancer patients with a better understanding of how those effects may be minimised with additional medicines may be expected to concord with their support medication. This decision might be reinforced by the knowledge that poor tolerance of chemotherapy might lead to dose reductions, treatment delays and possible disease progression or recurrence.

Whilst the association between the patient's level of understanding and treatment concordance cannot be assumed from this research, it is clear that the connection between the two concepts is becoming increasingly important to understand.

This study highlighted that further work needs to carried out on the association between level of patient understanding of therapy and treatment concordance. This is particularly important where the patient is being empowered to recognise the side effects of one set of medications (the chemotherapy) and self-treat by selection from a range of other medications (chemotherapy support medication). At this stage, it is sufficient to conclude that pharmacy input achieves greater understanding of these aspects in breast cancer patients.

### Chemotherapy delays and dose reductions

The pharmacy technician-led clinic produced significantly fewer treatment delays and dose reductions in this study. In most cases, delay was due to poor tolerance, which resulted in a dose reduction. The most common reason for delay was uncontrolled nausea and vomiting and there were notably more patients in the control arm (17) compared to the clinic arm (6). This is one area where the clinic appeared to be making an impact in terms of proper use of support medication, particularly with regard to use of appropriate antiemetic medication. Patients receiving adjuvant therapy might be expected to suffer from nausea and vomiting to a greater extent than patients receiving therapy for metastases and there were more of the latter in the clinic arm of the trial. In some cases, nausea and vomiting had been so severe that hospital admission was necessary to allow resultant dehydration to be resolved before administration of further chemotherapy. There were also more patients on regimens with greater emtogenicity in the clinic arm, notably FEC combinations (see Table 1).

More patients receiving trastuzumab monotherapy were randomised into the control rather than the clinic arm of the trial (13 *vs* two patients). Although this is a monoclonal antibody, emesis has been observed in our clinics and indeed is featured in the Summary of Product Characteristics. The Sussex Cancer Network (referred to above) advises in its standard treatment proforma for the drug that metoclopramide should be provided for all patients and that treatment should be subject to the same scrutiny as other drugs used in cancer chemotherapy. This was the case in our clinic where all therapies requiring support medication were included. Only one drug is recommended for support medication in this case, compared to greater numbers for other chemotherapy regimens encountered, reducing the potential for confusion. It is likely that the imbalance of trastuzumab numbers would decrease the impact of the pharmacy technician-led clinic rather than enhancing it if the numbers were the other way round. The imbalance is an artefact of strict randomisation.

Modern antiemetics, if used correctly, should result in fewer cases of delayed nausea and vomiting, but if patients do not know or cannot remember which ones to use and when, then their value is compromised. The efficacy of other oral treatments that patients may be taking may also be diminished due to impaired absorption and consequent sub therapeutic levels; for example in our study there were cases of neutropenic sepsis that may have been prevented had the oral antibiotic patients were taking been absorbed adequately. This study did not assess whether the antibiotic had actually been taken or mistaken for another support medication. A more in-depth study would be needed to determine the contribution of non-compliance with support medication to hospital admission of breast cancer patients for serious sequellae such as these.

### Drug interactions

Highlighting potential and existing drug interactions is an important part of any patient – carer interaction and a primary outcome of many studies of pharmacy and pharmacy technician drug history taking conducted in hospital settings.

Due to the shared care agreement that currently exists for oncology patients in the study area, drug histories are not taken routinely for cancer patients; this study has highlighted the difference the systematic taking of drug histories can make. Forty-seven of the 139 patients who participated in the study had potential drug interactions highlighted compared to none before the study began.

Most potential drug interactions were reported between GP prescribed medication and chemotherapy support medication rather than chemotherapy itself. The majority of these involved dexamethasone with NSAIDs, antidiabetic agents, anticonvulsants and antihypertensive agents.

### Chemotherapy support medication supplies

Effective medicines management is an important contributor to the quality of healthcare, which provides patient-focussed care based on need. In recent years, the Government has invested millions of pounds in developing medicines management across the NHS. Research has shown this strategy to produce financial and other benefits such as improved quality of patient care, more efficient dispensing, patient self-administration, medicines review and continual patient education. It has been shown that the concept of medicines management can be applied successfully to most areas and intuitively, should be of particular benefit when standard treatment regimens that require repeat dispensing are used.

In this study, medicines management and medication review in the pharmacy technician-led clinic reduced significantly the number of support medications required. This in turn equated to marked cost savings and a reduction in pharmacy dispensing time.

### Prescription interventions

The nature of a shared care agreement between two NHS Trusts does not always allow for efficient communication; for example in this case, two discrete sites are involved and the oncologist provides a visiting service from his base at one site to the other. Prescription issues often occur during periods when it is most difficult to resolve them quickly, for example when workload is high and after busy outpatient clinics when the oncologist is elsewhere. In this study, the pharmacy technician-led clinic led to a significant reduction in pharmacy time spent resolving prescription issues at the outpatient clinic.

In both study arms, the most common reported problem was that additions to chemotherapy support medication were unclear to staff. Clarification in the clinic when all healthcare team members are present (oncologist, nurse, and pharmacy staff) is of clear benefit to achieving the right patient prescription.

### Overall costs of the scheme

Cost analysis of the scheme (see results above) indicates that the initial, one-off payments for technician training (£1955.00) are more than covered by a projected annual saving of £15706.67 in terms of technician time saved (£1499.27) and reductions in dispensing unnecessary medicines (£14207.4). Clearly the actual annual amount will depend on patient throughput and complexity of their regimens, but this result does provide another indication that the approach is worthwhile.

## CONCLUSION

This study has demonstrated that breast cancer patients at Worthing Hospital had a significantly better understanding of their chemotherapy support medication after attending the pharmacy technician-led clinic compared to those patients who did not.

In addition, the study showed that for these patients there were highly significant reductions in the number of chemotherapy delays and dose reductions and the amount of repeat dispensing of chemotherapy support medication, resulting in a significant reduction in dispensary time and expenditure on medicines. There was also a highly significant reduction in pharmacy time spent resolving prescription issues at the point of dispensing.

We propose to extend the clinic to all breast cancer patients at Worthing Hospital and investigate its application to other specialities. A refinement to the process could include the amalgamation of the initial nursing assessment of the patient with the pharmacy technician-led clinic.

Chemotherapy support medications are commonly standard treatments employed for standard regimens with standard directions. Pre-labelled TTO packs could be available from the pre-assessment clinic under a Patient Group Direction after the pharmacy technician has taken a drug history and conducted a medication review to establish what is required, thus saving valuable dispensary time. The availability of a suitably qualified and accredited pharmacist supplementary prescriber in the pharmacy technician-led clinic could mean that a full service on chemotherapy support medication could be provided in the clinic without recourse to the dispensary. This is an attractive area for future research.

This study showed that as well as providing a better understanding of drug treatments, pharmacy technician-led clinics are also a prime opportunity to apply effective medicines management and medication review.

## Figures and Tables

**Table 1:** Nature of the chemotherapy regimes and treatment modes used in the control and clinic arms of the study

**Regimen**	**Control arm (no. of patients)**	**Clinic arm (no. of patients)**
Adjuvant FEC 60 mg m^−2^	8	15
Adjuvant EC 60	6	5
Adjuvant paclitaxel	5	—
Adjuvant docetaxel	—	2
Adjuvant FEC 75 mg m^−2^	17	26
Adjuvant AC	—	1
Adjuvant EC 75	—	3
Neo-adjuvant FEC 75	1	2
Metastatic epirubicin+docetaxel	2	—
Metastatic docetaxel	10	10
Metastatic trastuzumab	13	2
Metastatic vinorelbine	3	2
Metastatic EC 60	3	—
Metastatic capecitabine	2	—
Metastatic docetaxel and trastuzumab	—	2
		

**Chemotherapy cycle number**	**Control arm**	**Clinic arm**
2	53	53
3	15	13
4	2	4

A=Adriamycin (doxorubicin); C=cyclophosphamide; E=epirubicin; F=fluorouracil.

**Table 2:** Main outcome measures to demonstrating the impact of the pharmacy-led clinic (clinic arm) compared to control

**Feature**	**Control arm *n* (%)**	**Clinic arm *n* (%)**	**Statistical inference**
*Primary outcome measure: patients’ understanding of their medication*			
Number of patients (*n*)	69	69	
Mean baseline score	2.942	2.922	
Mean follow-up data score	2.965	5.417	
Difference between mean baseline and follow-up scores (95%CI)	0.023 (−0.228–0.182)	2.495 (2.165–2.826)	
*P*	0.822	<0.001	
			
*Secondary outcome measures*			
Number of patients having chemotherapy delays	25 (36.2)	8 (11.6)	*χ*^2^=11.51, *P*=0.001
Number of patients having chemotherapy dose reductions	21 (30.4)	7 (10.1)	*χ*^2^=8.78, *P*=0.003
Total number of patients with drug interactions recorded	20 (28.6)^a^	27 (39.1)	*χ*^2^=1.731, *P*=0.188
Median number of items of support medication supplied per patient per course	5^a^	2	*P*<0.001, 95%CI=1–3
Mean cost of items supplied per patient per course (+/− s.e.)	£26.7 (+/−4.7)^a^	£10.2 (+/−1.5)	*t*=3.34, *P*<0.001
Number of patient prescriptions requiring intervention	32 (45.7)	21 (30.4)	*χ*^2^=3.439, *P*=0.064
Mean time per patient spent resolving prescription issues (minutes)	9.9	1.8	Difference (95%CI)=8.1 (5.05–11.04), *P*<0.001

a One control subject was included at baseline, making *n*=70 for this calculation.
